# Development of a long non-coding RNA signature for prediction of response to neoadjuvant chemoradiotherapy in locally advanced rectal adenocarcinoma

**DOI:** 10.1371/journal.pone.0226595

**Published:** 2020-02-05

**Authors:** Lorenzo Ferrando, Gabriella Cirmena, Anna Garuti, Stefano Scabini, Federica Grillo, Luca Mastracci, Edoardo Isnaldi, Ciro Marrone, Roberta Gonella, Roberto Murialdo, Roberto Fiocca, Emanuele Romairone, Alberto Ballestrero, Gabriele Zoppoli

**Affiliations:** 1 Department of Internal Medicine, Università degli Studi di Genova, Genova, Italy; 2 IRCSS Ospedale Policlinico San Martino, Genova, Italy; 3 Department of Integrated Surgical and Diagnostic Sciences, Università degli Studi di Genova, Genova, Italy; University of Nebraska Medical Center, UNITED STATES

## Abstract

Standard treatment for locally advanced rectal adenocarcinoma (LARC) includes a combination of chemotherapy with pyrimidine analogues, such as capecitabine, and radiation therapy, followed by surgery. Currently no clinically useful genomic predictors of benefit from neoadjuvant chemoradiotherapy (nCRT) exist for LARC. In this study we assessed the expression of 8,127 long noncoding RNAs (lncRNAs), poorly studied in LARC, to infer their ability in classifying patients’ pathological complete response (pCR). We collected and analyzed, using lncRNA-specific Agilent microarrays a consecutive series of 61 LARC cases undergoing nCRT. Potential lncRNA predictors in responders and non-responders to nCRT were identified with LASSO regression, and a model was optimized using k-fold cross-validation after selection of the three most informative lncRNA. 11 lncRNAs were differentially expressed with false discovery rate < 0.01 between responders and non-responders to NACT. We identified lnc-KLF7-1, lnc-MAB21L2-1, and LINC00324 as the most promising variable subset for classification building. Overall sensitivity and specificity were 0.91 and 0.94 respectively, with an AUC of our ROC curve = 0.93. Our study shows for the first time that lncRNAs can accurately predict response in LARC undergoing nCRT. Our three-lncRNA based signature must be independently validated and further analyses must be conducted to fully understand the biological role of the identified signature, but our results suggest lncRNAs may be an ideal biomarker for response prediction in the studied setting.

## Introduction

To date, surgery represents the gold standard in locally advanced rectal adenocarcinoma (LARC), together with neoadjuvant chemoradiotherapy (nCRT) [1]. Preoperative treatment has the potential to reduce tumor size and local risk of recurrence, partially or completely block tumor cancer invasion, and increase tumor resection rate [2]. Nevertheless, tumor response to nCRT is not uniform and shows a wide range of responsiveness, ranging from pathological complete response (pCR) to complete resistance. Moreover, surgical outcome can be affected by nCRT [3]. Assessment of nCRT effectiveness introduces a substantial clinical dilemma about the benefit and the risks of the administration of nCRT in LARC. The ability to predict response to nCRT is crucial to optimize timing of surgery, avoiding side effects and toxicity of nCRT, and to reduce operative morbidity and surgical complications [4–5].

Several biomarker studies over the last few years have approached the issue of sensitivity to nCRT from the point of view of response prediction [6]. To this purpose, coding gene expression microarrays, as well as transcriptome sequencing or other methods aimed at assessing various molecular species have been employed [7–8–9–10]. While in general individual studies often report promising results, two issues have not been resolved: the overall suboptimal capability of coding transcripts and non-coding ones such as microRNAs (miRNAs) to act as predictive markers with medical utility [11], and the lack of focus on more recently described, less studied, and potentially promising alternative categories of molecular species, such as long noncoding RNAs (lncRNAs), which exhibit interesting property of degradation stability in tissues and fluids [12].

In our work, we aimed to assess the relative abundance of more than 8,000 lncRNAs using dedicated microarrays in a large, prospective, consecutively collected cohort, seeking to identify novel promising predictive biomarkers for nCRT in LARC.

## Patients and methods

### Patients and material collection

For the present work, we consecutively collected pre-treatment tumor specimens from patients diagnosed with LARC, clinical stage II (T3-T4, N-) or III (any T, N+), located within 12 cm from the anal verge as determined by rigid or flexible proctoscopy. Clinical staging was determined using endoscopic ultrasound (EUS) and pelvic nuclear magnetic resonance (NMR) for local disease assessment, and by whole-body computer assisted tomography (CAT) scan to exclude distant metastases before the administration of the neoadjuvant treatment. All patients underwent a fixed-dose regimen based on the concomitant administration of capecitabine (825 mg/m^2^) and radiation treatment (50.4 Gy) divided in 28 fractions over 5 weeks. 5–6 weeks after preoperative treatment, patients were restaged for resectability and limited disease using EUS, NMR, and CAT scan. Surgery was performed 10–12 weeks from the completion of neoadjuvant therapy. Criteria for exclusion from the present study were: inability or refusal to comply with treatment, refusal to consent or withdrawal of consent for biopsy and/or subsequent analyses, post-treatment radiological diagnosis of distant disease, completion of less than 85% of the planned treatment, intermediate pathological regression score according to Dworak tumor regression grading [13]. The latter exclusion criterion was motivated by our *a priori* assumption that biologically relevant differences could be better observed when comparing the extremes of response to nCRT.

Two pre-neoadjuvant treatment tumor specimens were collected: the first specimen was formalin-fixed and paraffin-embedded for histopathological diagnostic confirmation, and the second underwent Optimal Cutting Temperature embedding, freezing over nitrogen vapors, and storage at -80°C for research purposes.

For the sake of our analyses, we considered as “major responder” (responders) patients those with Dworak score equal to 3–4, “minor responder” (non-responders) patients those with Dworak equal to 0–1, and intermediate responders, when Dworak score was 2.

A written informed consent for translational studies was obtained from all patients, and our Internal Ethics Committee approved the present study.

### cDNA microarrays analyses

Samples were thawed and processed for DNA and RNA extraction using Norgen Biotek Corp. RNA/DNA Purification Plus kits according to Manufacturer’s specifications.

cDNA amplification was performed starting from 100 ng of total RNA using TransPlex Whole Transcriptome Amplification Kit (Sigma-Aldrich Co.). cDNA was then purified using the QIAquick PCR purification kit (Qiagen) and relative quantity was assessed using a NanoDrop ND-1000 Spectrophotometer (Thermo Scientific Inc.) as described previously [14].

RNA was labeled according to Manufacturer’s instructions and hybridized to Agilent SurePrint G3 Human Gene Expression v2 8x60K microarrays, which include 8,127 lncRNA-dedicated probes as well as probes for 20,560 known coding transcripts. Hybridized chips were then scanned on an Agilent Technologies Inc. G2565C device.

### Statistical analyses and power considerations

Quality control was evaluated using both the proprietary software Agilent SureScan Microarray Scanner® and the *arrayQualityMetrics* BioConductor package [15]. Expression data were extracted from image files using the Feature Extraction Software v10.7 (Agilent Technologies). Pre-processing and normalization were performed in the R environment for Statistical Computing using *limma* [16] as previously described, and scanning batch was considered as a random covariate for preliminary quality assessment. Since no meaningful differences in results were observed with or without the scanning batch variable, such covariate was later excluded from our analyses. We performed background correction based on negative control probes and quantile-normalization using both negative and positive controls [17]. Samples were normalized both within and between arrays, according to the standard pipeline outlined for Agilent microarrays in [18]. Subsequently, normalized expression data were log2-transformed and control probes were removed. Within-array replicate probes were replaced with their average to reduce expression variability and to enhance computational robustness.

LASSO regression was applied in order to select a subset of relevant lncRNAs, reducing dimensionality of variable set and improving interpretability. For such analysis we used the *glmnet* package [19] with default settings, and leave-one-out cross-validation technique. Selected variables were then formally assessed for statistical significance using Welch’s t-test, and p-values were corrected for multiple testing using the Benjamini-Hochberg false discovery rate (FDR) method [20]. To further define a robust yet simple classifier, we heuristically resorted to including in our final model the three variables with highest importance, as resulting from the well-established polynomial support vector machine [21] implemented in the *caret* package [22] with default settings and 5-fold cross-validation to limit bias. Importance was assessed as described in [22] where relevance of predictors is individually determined calculating its relative receiver operating characteristic (ROC) area. Model selection was performed using *caret* package, and the *pROC* package was used to plot ROC curves.

We based the number of analyzed samples on the following considerations. In our Center’s experience, ~35% of patients achieve a regression score 3–4, ~50% undergo treatment resulting in a Dworak regression score = 2, and 25% of patients have a score 0–1 at post-surgical pathological examination. With those proportions in mind, assuming a maximum transcript standardized fold change between groups of ~2, on the ground that we assessed 8,127 lncRNAs, and excluding as stated above IR patients, we foresaw that we would need to collect specimens from at least 58 consecutively treated patients in order to have at least 12 non-responders and 17 responders (and–theoretically– 29 intermediate response cases). With this scenario, we would be able to perform two-group classification analyses with an expected tolerance from the best possible classifier of 0.10, as defined in [23].

## Results

### Demographics and clinical features

**Fig 1:**
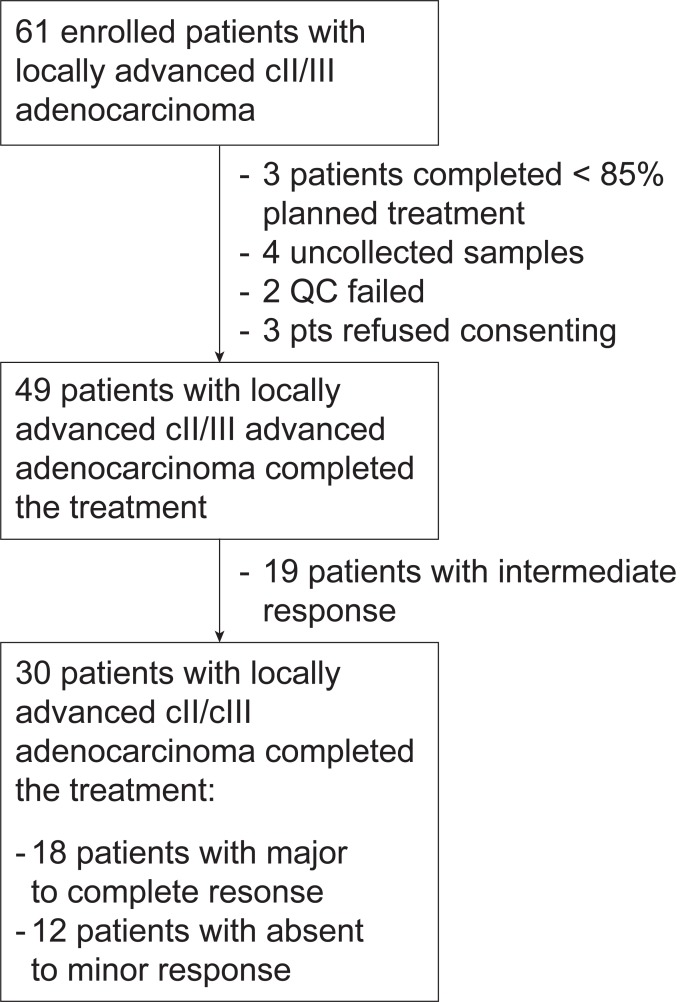
Consort-like flow diagram of the study.

Out of 61 initially screened cases, 3 patients refused to consent, 3 patients did not complete at least 85% of planned treatment, 4 samples were not collected, and 2 samples failed quality control. Of the remaining set of 49 patients, 12 were classified as non-responders and 18 as responders by histopatologic examinations according to TRG. Cases with intermediate response (TRG = 2, N = 19) were removed from final analyses to simplify interpretation of our results and to highlight biological difference in the extremes of the response range (Fig 1). All samples used to build our classification model passed quality check control process. Patients’ characteristics are summarized in Table 1. The median age was 68 years (IQR: 60–73 years) in line with the median age of hospitalized patients in our Center and all patients completed the preoperative treatment as per protocol specifications. There were no significant differences between the two classes in terms of clinico-pathologic parameters as assessed by Fisher tests or Welch tests as appropriate.

**Table 1:** Clinico-pathologic characteristics of 30 patients with LARC.

Median age	68 (IQR, 61–73)
TIL	
Yes	6 (20%)
No	24 (80%)
Node Positive	
Yes	26 (87%)
No	4 (13%)
Sex	
Male	15 (50%)
Female	15 (50%)
Tumor regression grade	
0	2 (7%)
1	10 (33%)
3	9 (30%)
4	9 (30%)

### lncRNAs stratify LARC patients into responders vs. non-responders

**Fig 2:**
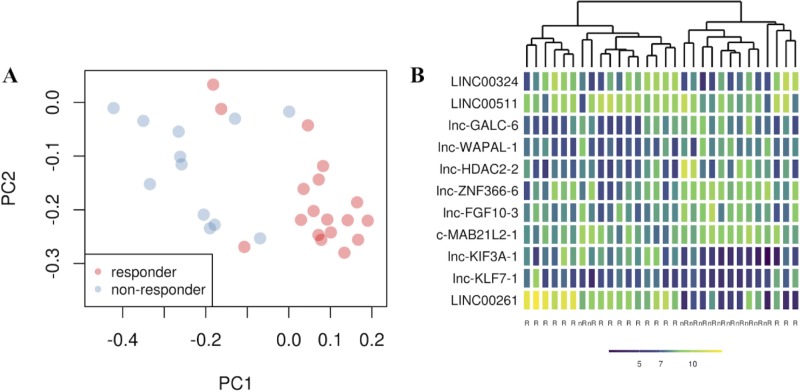
(A) principal component analysis shows the position of each sample on a bi-dimensional graph. The x- and y-axes represent the first (PC1) and the second (PC2) principal component respectively. Red dots are responder patients, whereas blue dots are non-responder patients as defined in the Methods section. (B) Heatmap plot identifies gene expression patters of 11 lncRNAs in 30 LARC samples. The x-axis represents the class of samples (nr = non-responder, R = responder). The y-axis represents transcript expression. Each cell shows a color that ranges from dark blue to yellow. The darker the blue is, the lower the expression is and vice versa.

To identify a genomic signature of response to nCRT, we analyzed the expression of 8,127 lncRNAs, represented in the microarrays we used. Using LASSO regression [24] for initial variable extraction, we identified an initial list of 11 differentially expressed lncRNAs (Table 2). Out of 11, 5 lncRNAs were over-expressed, while the remaining genes were under-expressed in responders. After evaluating differentially expressed lncRNAs, we determined the efficacy of the identified molecules in separating responders and non-responders. For this purpose, we conducted a principal component analysis (PCA) with the 11 lncRNAs found by LASSO, which resulted in a surprisingly effective separation of the two classes (Fig 2A).

**Table 2:** lncRNAs differentially expressed between responders and non-responders.

Probe name	Gene symbol	Genebank	FC	FDR
A_32_P23125	LINC00261	NR_001558	6.59	0.0018
A_21_P0014615	lncKIF3A-1	-	3.95	0.0016
A_23_P362191	LINC00324	NR_026951	3.63	0.0022
A_33_P3310649	lncKLF7-1	TCONS_00003489	2.95	0.0003
A_33_P3741022	LINC00511	NR_033876	1.81	0.0195
A_21_P0007008	lncWAPAL-1	TCONS_00018561	0.59	0.0081
A_21_P0012985	lncFGF10-3	TCONS_l2_00023730	0.55	0.0033
A_33_P3209326	lncMAB21L2-1	AK096995	0.51	0.0007
A_21_P0008471	lncGALC-6	TCONS_00022822	0.47	0.0018
A_21_P0004412	lncZNF-366-6	TCONS_00010375	0.47	0.0022
A_19_P00317053	lncHDAC2-2	TCONS_00012259	0.36	0.0018

Gene expression levels of these lncRNAs were used to perform hierarchical clustering analysis (Fig 2B). Unsupervised hierarchical analysis was consistent with PCA in separating responders and non-responders. These results showed that selected lncRNAs had the potential to classify the response to nCRT in our LARC dataset.

### Differentially expressed lncRNAs correspond to relevant molecules in cancer

**Fig 3:**
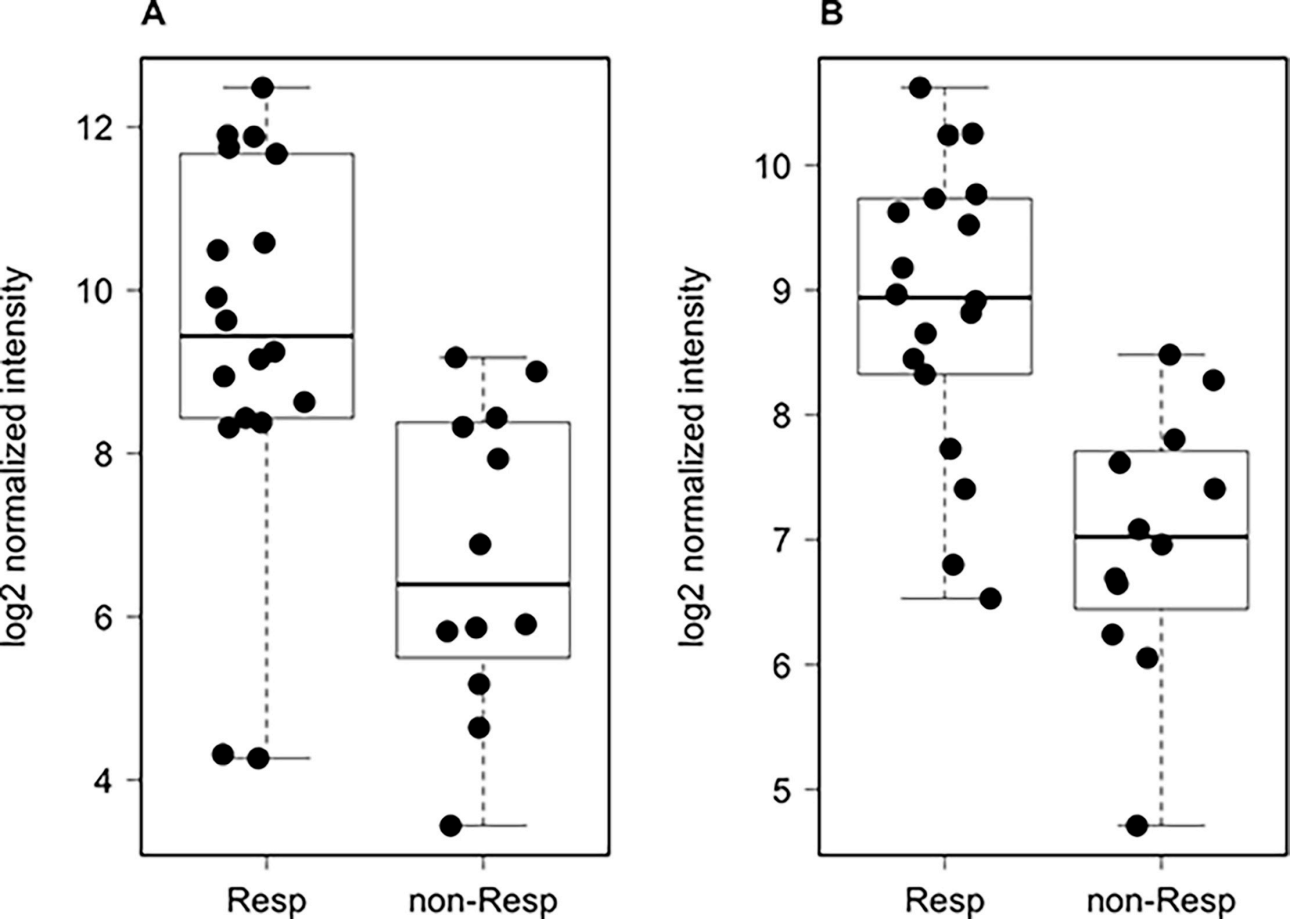
Comparison of gene expression profiles of the two transcripts, (A) LINC00261 and (B) LINC00324. Y-axis represents log2-transformed normalized expression, whereas x-axis show the responder and non-responder groups.

While many lncRNAs still miss a known role in cancer, we observed that several of the lncRNAs identified by our analysis have been independently described in association with neoplastic disorders. Of special interest, LINC00261 exhibited a 6.6-fold change between responders and non-responders (FDR = 0.0018, Fig 3A), and was recently described for its role in sensitization to chemotherapy in colorectal adenocarcinoma models [25], potential prognostic role in lung cancer [26], and progression regulation in endometrial carcinoma [27]. Moreover, LINC00324, with a fold-change = 3.6 (FDR = 0.0022, Fig 3B), and LINC00511 (fold-change = 1.81, FDR = 0.0196), have been characterized in comprehensive efforts to understand the functions of such molecules in cancer [28], potentially acting as bona fide oncogenes [29]

### Three lncRNAs exhibit great accuracy as a predictive signature for response nCRT

**Fig 4:**
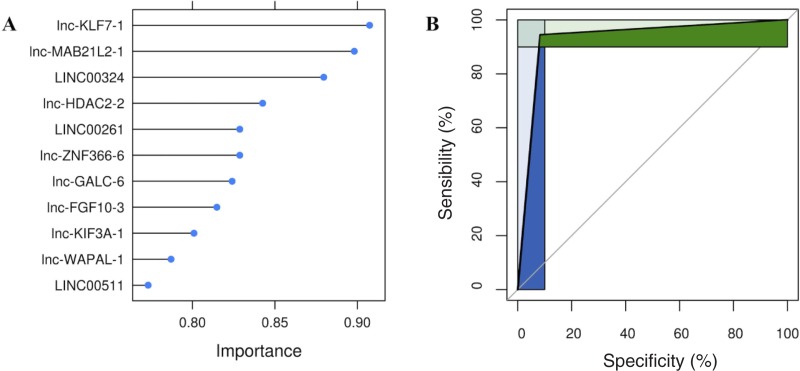
(A) needle plot of the SVM based on polynomial kernels. The graph shows feature importance calculated determining AUC for each predictor. The ranking allows to assess the minimum number of features to design an optimized simpler classifier. (B) ROC curve shows the diagnostic performance of lncRNAs signature. Green and blue areas represent partial area under the curves (pAUC), corresponding to clinically relevant regions of sensibility and specificity.

In order to increase the robustness of a lncRNA-based classifier in spite of the relatively small size of the set we used, we selected the final variables for our model by taking into account their relative importance according to the SVM algorithm used (Fig 4A). We thus decided to use lnc-KLF7-1, lnc-MAB21L2-1, and LINC00324, reasoning that the addition of further variables to our final model would marginally increase classification ability at the cost of undesirable overfitting.

Amazingly, the use of only three variables resulted in an extremely high overall sensitivity and specificity, with small confidence intervals: 0.91 and 0.94 respectively, with an area under the curve (AUC) of our ROC curve = 0.93 (CI: 0.83–0.99, Fig 4B).

## Discussion

Over the past decade, translational research in the field of LARC has been focused on the issue of identifying reliable markers to inform clinicians on the potential of nCRT to result in a significant benefit for patients. To this purpose, several microarray-based and next-generation-sequencing (NGS) studies explored the expression of coding transcripts and microRNAs, with the aim of finding a medically useful predictive molecular signature [30, 31, 32]. While showing biologically meaningful associations, these studies could hardly generate predictors with an immediate transferability to clinical practice. In the present work, we explored the expression of a still poorly explored molecular species in the context of LARC, lncRNAs in association with standard nCRT. Our findings show that not only lncRNAs are differentially expressed in a meaningful way in responder vs. non-responder patients, but also that such category of molecules is potentially able to stratify cases with surprisingly high accuracy. From our experiments, we indeed observed lncRNAs that have been independently associated with tumorigenesis, prognosis, and outcome in several tumor subtypes including colorectal cancer [25, 26, 27]. In particular, LINC00261 appears repeatedly in the scientific literature for its functional association with several neoplastic and proliferative disorders [33, 34, 35, 36, 37], and its identification as a highly differentially expressed molecule in LARC according to nCRT deserves special attention for its potential in translational research. Indeed, there is an increasingly evidence that lncRNAs could drive cancer phenotypes, interacting with different molecular species including proteins, coding transcripts, and even DNA structures [38].

The most intriguing finding of our work, however, lies in the accuracy that a three-molecule lncRNA-based signature may have to predict response to nCRT in LARC. Actually, despite the small size of our sample set, we were amazed by the predictive power that our predictive model could achieve. If confirmed in larger, independent case sets, a lncRNA-based classifier such as the one we developed could outperform larger predictive models based on other, more commonly examined molecular species. Furthermore, lncRNAs seem to exhibit remarkable stability in tissues [39], thus providing an attractive molecular species for studies aimed at clinical transferability.

We are aware that our study has several limitations, *in primis* the lack of functional insights into our findings. Furthermore, the size of our cohort, albeit acceptable according to power considerations, is still limited and requires an independently obtained set to confirm our findings. We plan to deepen, with future functional analyses, the biological role of our signature, in order to determine if it can represent a reliable predictive marker for response to nCRT in LARC.

## Conclusion

To the best of our knowledge, we have for the first time showed in an appropriately sized, consecutively accrued, prospectively treated cohort that lncRNA expression assessment in the context of response prediction to nCRT in LARC is not just informative, but indeed extremely promising. In a context where biomarkers are currently lacking and lack of response to nCRT is not only associated with worse outcome, but also with less satisfactory surgical results [40, 41], our work represents a novel and attractive venue for clinical research.

### Clinical practice points

- lncRNAs, a poorly studied molecular category in locally advanced rectal adenocarcinoma (LARC), are differentially expressed according to response to neoadjuvant chemoradiotherapy (nCRT).
- We identified lncRNAs that differentiate between responder and non-responder patients from pretreatment biopsies.
- A three-lncRNA signature predicts response to nCRT in LARC with extremely high accuracy and shows promise for clinical transferability.

## Abbreviations

LARC: locally advanced rectal adenocarcinoma
nCRT: neoadjuvant chemoradiotherapy
pCR: pathological complete response
miRNA: microRNA
lncRNA: long noncoding RNA
EUS: endoscopic ultrasound
NMR: nuclear magnetic resonance
CAT: computer assisted tomography
TRG: Dworak tumor regression grade
FDR: false discovery rate
ROC: receiver operating characteristic
AUC: area under the curve
SVM: support vector machine
